# Structural and Functional Determinants of γ-Secretase, an Intramembrane Protease Implicated in Alzheimer’s Disease

**DOI:** 10.2174/138920207783769521

**Published:** 2007-12

**Authors:** Patrick C Fraering

**Affiliations:** Brain Mind Institute and School of Life Sciences, Swiss Federal Institute of Technology (EPFL), CH-1015 Lausanne, Switzerland

## Abstract

Alzheimer’s disease is the most common form of neurodegenerative diseases in humans, characterized by the progressive accumulation and aggregation of amyloid-β peptides (Aβ) in brain regions subserving memory and cognition. These 39-43 amino acids long peptides are generated by the sequential proteolytic cleavages of the amyloid-β precursor protein (APP) by β- and γ-secretases, with the latter being the founding member of a new class of intramembrane-cleaving proteases (I-CliPs) characterized by their intramembranous catalytic residues hydrolyzing the peptide bonds within the transmembrane regions of their respective substrates. These proteases include the S2P family of metalloproteases, the Rhomboid family of serine proteases, and two aspartyl proteases: the signal peptide peptidase (SPP) and γ-secretase. In sharp contrast to Rhomboid and SPP that function as a single component, γ-secretase is a multi-component protease with complex assembly, maturation and activation processes. Recently, two low-resolution three-dimensional structures of γ-secretase and three high-resolution structures of the GlpG rhomboid protease have been obtained almost simultaneously by different laboratories. Although these proteases are unrelated by sequence or evolution, they seem to share common functional and structural mechanisms explaining how they catalyze intramembrane proteolysis. Indeed, a water-containing chamber in the catalytic cores of both γ-secretase and GlpG rhomboid provides the hydrophilic environment required for proteolysis and a lateral gating mechanism controls substrate access to the active site. The studies that have identified and characterized the structural determinants critical for the assembly and activity of the γ-secretase complex are reviewed here.

## INTRODUCTION

Alzheimer’s disease (AD) is the most common form of neurodegenerative diseases in humans, leading to nerve cell death and tissue loss throughout the brain, therefore impairing memory, reasoning and behavior and leading ultimately to complete social dependence and death. During the past two decades, a working hypothesis known as the ‘amyloid-β cascade hypothesis’ has been developed suggesting that the progressive accumulation/oligomerization/aggregation of amyloid-β peptides (Aβ) in brain regions subserving memory and cognition, including the frontal cortex and the hippocampus, is the primary cause of neurodegeneration and cell death in AD [1]. Compelling genetic evidence supporting this hypothesis came in 1991, when missense mutations in the gene that encodes the Aβ precursor protein (APP) were found to cause AD in certain families [2-4].

Aβ is formed from amyloid-β precursor protein (APP) through two protease activities [5]. First, the membrane tethered aspartyl protease β-secretase (BACE 1, [6]) cleaves APP at the Aβ N-terminus (β cleavage site), resulting in a soluble form of APP (sAPPβ; Fig. (**1**)) and a 99-residue (~12kDa) C-terminal fragment (C99; Fig. (**1**)). This C-terminal fragment undergoes subsequent processing by γ-secretase (γ cleavage site) resulting in the formation of Aβ peptides of various lengths (39-43 amino-acid residues), with the two primary forms being the 40- and 42- amino acid variants, Aβ40 and Aβ42. Although Aβ42 accounts for only 10% of total Aβ secreted from cells (Aβ40 predominants), Aβ42 is the major protein component of amyloid plaques [7-9] and aggregates much more rapidly than Aβ40 *in vitro* [10]. An alternative non-amyloidogenic processing pathway of APP molecules involves its cleavage within the Aβ region (α  cleavage site), by another member of the secretase family, the α -secretase metalloproteases ADAM10 and tumor necrosis factor α  (TNF-α )-converting enzyme (TACE/ADAM17)  [11, 12]. Cleavage by α -secretase precludes the formation of Aβ and results in the release of a soluble form of APP (sAPPα ; Fig. (**1**)) and membrane retention of a 83-residue (~10kDa) C-terminal fragment (C83) (Fig. (**1**)). Subsequent cleavage of C83 by γ-secretase results in an N-terminally truncated and non-toxic Aβ protein of 3 kDa (p3; Fig. (**1**); [13]). In addition to APP processing, γ-secretase has been implicated in intramembranous cleavage of a whole set of type I membrane proteins, including Notch, the growth factor dependent receptor Erb-B4, the cell adhesion molecules N-cadherin, E-cadherin, CD44, and the neurotrophin co-receptor p75 (for a review, see [14]).

In cells, γ-secretase activity is associated with a high molecular weight multiprotein complex that is composed at least of four integral membrane proteins, presenilin (PS, 9 TMDs), Nicastrin (NCT, 1 TMD), PEN-2 (2 TMDs), and APH-1 (7 TMDs), with PS (and two conserved and crucial intra-membrane aspartate residues) constituting the catalytic component [16] and the other 3 proteins acting as essential cofactors and/or scaffold proteins necessary for the assembly and activation of the complex [17, 18]. NCT has further been reported to be involved in the recognition and binding of substrates [19] whereas PEN-2 triggers the endoproteolysis of PS into PS-NTF and PS-CTF during the maturation/activation of the complex [17]. Indeed, studies performed in *Drosophilia* and mammalian cells have shown that NCT, APH-1 and PEN-2 stabilize and promote the increased formation of mature PS N- and C-terminal fragments and γ-secretase activity, suggesting that PS heterodimers (NTF/CTF), NCT, APH-1 and PEN-2 are both necessary and sufficient for γ-secretase activity [17, 18]. The reconstitution of γ-secretase activity in *S. cerevisiae* (which lacks γ-secretase) by expressing just these four components further supports this conclusion [20]. The subsequent high-grade purification of the human γ-secretase complex from CHO cells that stably overexpress human PS1, APH-1 and PEN-2 (designated γ-30 cells) accompanied by a detailed mass spectral analysis of all the proteins found in the catalytically active fraction provided further compelling evidence that PS NTF/CTF, NCT, APH-1 and PEN-2 are the components of active γ-secretase [21]. Because understanding the workings of γ-secretase is important for both biomedical research (because of its role in the generation of Aβ, γ-secretase has emerged as a key therapeutic target for AD) and basic biology (i.e. cell signaling and stem cells differentiation), structural information of the protease complex is needed to gain functional insights into its biochemistry. Electron microscopy (EM) and single particle image analysis on the enzyme purified from the γ-30 cells revealed the presence of a low-density interior chamber and apical and basal pores that could allow the entry of water molecules (required to accomplish peptide bond hydrolysis) and their sequestration from the lipid surround by the proteinaceous microenvironment provided by the 19 transmembrane domains [22]. This first 3D structure of the complex also provided plausible explanations about how γ-secretase releases its cleavage products into distinct subcellular compartments [22]. Despite this progress, the exact spatial organization and functional contribution of each γ-secretase subunit to the enzymatic activity remain largely unknown. 

γ-Secretase is the founding member of an emerging class of *i*ntramembrane-*cl*eav*i*ng *p*roteases (I-CliPs) that includes site 2 protease (S2P) cleaving membrane-anchored transcription factors involved in cholesterol biosynthesis [23], Rhomboids responsible for releasing epidermal growth factor in *Drosophila* [24] and signal peptide peptidase (SPP), a PS-like protein that requires two conserved aspartates to cleave remnant signal peptides left in the membrane after generation by signal peptidase [25]. Like γ-secretase, S2P, Rhomboid, and SPP all hydrolyze their respective substrates within their TM regions, and the residues responsible for catalysis are either intramembranous or at the predicted membrane-cytosol interfaces of the proteases. Recently, atomic structures of the GlpG rhomboid protease from *Escherichia coli* were reported [26-28]. Although GlpG rhomboid protease from *E. coli* and γ-secretase are unrelated by sequence or evolution, they seem to share common functional and structural mechanisms explaining how they catalyze intramembrane proteolysis. 

### The Minimal Set of Components to Constitute γ-Secretase Complex

#### 1. Presenilin 1 and Presenilin 2: Catalytic Components of the γ-Secretase Complex

In 1995, independent groups identified genetic linkage and mutations within *PSEN1* (chromosome 14, 14q24.3) and *PSEN2* (chromosome 1, 1q42.2) genes in several early onset familial Alzheimer’s disease (FAD) kindreds [29-31]. So far, 165 different mutations in *PSEN1* *(www.molgen.ua.ac.be/AD Mutations/default.cfm?MT=1&ML=1&Page=MutByQuery& Query=tblContexts.GeneSymbol%20In%20('PSEN1')&Selection=Gene%20In%20(PSEN1)&CFID=196010&CFTOKEN=37631420)*and 9 different mutations in *PSEN2* *( www.molgen.ua.ac.be/ADMutations/default.cfm?MT=1&ML=1&Page=MutByQuery&Query=tblContexts.GeneSymbol%20In %20('PSEN2')&Selection=Gene%20In%20(PSEN2)&CFID=196010&CFTOKEN=37631420)* have been identified to cause FAD. *PSEN1* and *PSEN2* encode 467 and 448 amino acid-long polytopic transmembrane proteins, termed presenilin 1 (PS1) and presenilin 2 (PS2), respectively. The sequence identity between these two highly conserved proteins is 67% [29].

##### Transmembrane Topology of Presenilin 1

Until now, several different models for the transmembrane domain (TMD) topology of PS1 have been proposed, including a 6-TMD model [32], a 7-TMD model (reviewed in [33], and a 8-TMD model topology with the N- and C- termini, and a hydrophilic loop domain between TMD6 and TMD7 facing the cytosol, which has been, until very recently, widely accepted by the Alzheimer’s community [34-36]. However, five very recent computational and experimental studies have demonstrated that the N-terminal domain of PS1 is cytosolic, the C-terminal domain of PS1 is extracytoplasmic, and that PS1 adopts a 9-TMD topology [37-41] as depicted in Fig. (**2**). Further evidence supporting the latter model comes from the observation that signal peptide peptidase (SPP), one of the five PS homologues identified in the human genome based on sequence conservation, adopts a 9-TMD topology, although with an opposite membrane orientation [42-44]. 

##### Domains of PS1 Critical for the Assembly and/or the Activity of the γ-Secretase Complex

###### “TMD1” Motif

A large deletion in PS1-NTF that removes the two first TMDs has been reported by two independent studies to cause reduced endoproteolysis and reduced γ-secretase activity [45, 46]. Brunkan *et al*. have used a mutational analysis approach and rescue assays in cultured PS1/PS2 knockout fibroblasts to investigate the sequence requirements in TMD1 of PS1 necessary to support both γ-secretase and presenilinase (endoproteolysis of PS into PS-NTF and PS-CTF) activities [47]. This study allowed for the identification of two distinct domains within TMD1 of PS1: (1) an N-terminal domain (residues 87 to 90) that is important for γ-secretase activity but not for presenilinase activity, and (2) a C-terminal domain (residues 95-98) that is essential for both enzymatic activities. Importantly, photoactivatable transition-state analog inhibitors targeting the active site of γ-secretase failed to bind to PS1 containing the V96F mutation, supporting the hypothesis that the residues 95-98 are required for proper active site conformation [47]. 

###### “NF” Motif (TMD4)

We recently demonstrated a physical interaction between PS1-NTF and PEN-2 by promoting the partial dissociation of the γ-secretase complex using dodecyl β-D-maltoside (DDM), a mild detergent [48].The DDM-resistant interactions identified in the dissociated complexes likely reflect the physical interactions existing in the active γ-secretase complex. Moreover, the use of an antibody targeting the extreme C-terminus of PS1 revealed that PS1-holoprotein can associate with PEN-2 in a manner independent of the other γ-secretase components NCT and APH-1, further supporting a direct physical interaction between PS1 and PEN-2 [48]. Two independent groupsrecently used the so called “Swap method” (replacement of specific TMDs of PS1 with TMDs of unrelated membrane proteins) for the analysis of the individual roles of PS1 TMDs for binding with PEN-2 [49, 50]. Both studies have identified a highly conserved consensus motif (XWNFGX, residues 202-207 in human PS1) among multiple organisms required and sufficient for PS1:PEN-2 interactions. Indeed, chimeric mutant PS1 swapped at TMD4 [49, 50] and PS1 NFGV to YIIL mutant [50] lack γ-secretase activity because of loss of interaction with PEN-2. Collectively, both studies have demonstrated that the NF motif in TMD4 of PS1 is critical for binding with PEN-2, thus promoting PS1 endoproteolysis and activation of γ-secretase. Furthermore, the same studies have shown that TMDs 1, 2, 3, 5 and 6 of PS1 are dispensable for interaction with PEN-2, but critical for γ-secretase activity [49, 50].

###### Exon 9 Hydrophobic Domain (between TMD6 & TMD7)

HumanPS1 is synthesized as a 43 kDa polypeptide that undergoes highly regulated endoproteolysis to generate stable 27 kDa N-terminal (PS1-NTF) and 16 kDa C-terminal (PS1-CTF) fragments in a 1:1 stoichiometry [51]. This endoproteolytic cleavage occurs within the large cytoplasmic loop connecting TMD 6 and 7, between amino acid residues M292 and V293 of human PS1 (or M298 and V299 of human PS2) in a highly hydrophobic domain known to be deleted in a PS1 variant that lacks amino acid residues 290-319 following a point mutation upstream of a splice acceptor site resulting in an in-frame deletion of exon 9 (PS1∆E9) [51-55]. This mutation has been identified in the PS1 gene in families with early onset AD and the corresponding PS1∆E9 protein is not subject to endoproteolytic cleavage [51, 52]. Specific mutations of the endoproteolytic cleavage sites of human PS1 (single mutants M292D and M292E, and double mutant M292D/V293K) and human PS2 (M298D/V299A) block or greatly reduce PS endoproteolysis [54-56]. Whether these mutations affecting PS endoproteolysis also affect directly γ-secretase activity remains unclear, but strong evidence supports the hypothesis that this endoproteolytic event is a critical step in the assembly and activation of γ-secretase (discussed below).

###### Aspartate 257 (TMD6) and Aspartate 385 (TMD7): The Core of an Aspartyl Catalytic Site

Different Calpain cystein protease inhibitors including MDL28170 (carbobenzoxyl-Val-Phe-alaninal), Calpain I, MG 132 (carbobenzoxyl-Leu-Leu-leucinal) and Calpeptin (carbobenzoxyl-Leu-norleucinal)have first been reported to reduce the amounts of Aβ and P3 secreted from different cell lines transfected with APP (carrying the Swedish mutations or not), while C-terminal APP intermediates accumulated within the cells, thus suggesting a direct inhibition of γ-secretase [57-60]. Moreover, these peptide aldehyde cystein protease inhibitors did not affect Aβ40 and Aβ42 with the same potency, suggesting two pharmacologically distinct proteolytic activities: a γ(40)-secretase and a γ(42)-secretase generating Aβ40 and Aβ42, respectively [59, 60]. It has also been reported that Pepstatin A, an inhibitor of the lysosomal aspartyl proteinase cathepsin D, inhibited the cleavage of APP-based substrates resulting in Aβ production [61, 62]. The latter data suggested that cathepsin D itself or a cathepsin D-like activity maybe γ-secretase – with the first hypothesis being rapidly ruled out by the observation that Aβ production and secretion was not affected in primary cultures of hippocampal neurons derived from cathepsin D knock out mice [63]. The first pharmacological evidence supporting an aspartyl protease mechanism for γ-secretase came with the finding that peptide analogues specifically designed based on the Ala713-Thr714 APP cleavage site leading to Aβ42 (substrate-based difluoro ketones that mimic an intermediate in aspartyl-protease catalysis) inhibit Aβ production in both cell-based and cell-free microsomal γ-secretase activity assays [64, 65]. Moreover, these inhibitors were not effective against calpain (cystein protease) but did inhibit cathepsin D (aspartyl protease), further supporting that γ-secretase is an aspartyl protease [65]. Next, two conserved transmembrane aspartate residues in PS1 have been identified to be essential for γ-secretase activity and PS endoproteolysis [16]. Indeed, mutation of Asp 257 (D257A in TMD6) or Asp 385 (D385A in TMD7) of human PS1 substantially reduce Aβ production and increase the amounts of the APP-based substrates of γ-secretase [16]. Moreover, biotinylated transition-state analogs designed to interact specifically with the catalytic aspartates of γ-secretase bind specifically and directly to PS1 (and PS2)-NTF and PS1 (and PS2)-CTF [66, 67]. Finally, PS-deficient (PS1-/- PS2 -/- double knockout) embryonic stem cells are completely devoid of γ-secretase activity [68, 69]. When taken together, these findings support the notion that PS forms the catalytic center of γ-secretase with two transmembrane aspartate residues (Asp 257 and Asp 385 in human PS1, and Asp 263 and Asp 366 in human PS2) forming the catalytic dyad of the enzyme. 

###### “GxGD” Motif (TMD7)

PS, signal peptide peptidase (SPP) and presenilin homologs/SPP-like proteins (PSHs/SPPL) contain a highly conserved GxGD sequence motif (residues 382-385 in human PS1) including the C-terminal active site aspartate (TMD7) [70, 71]. The glycine 384, which is immediately adjacent to the critical aspartate at residue 385 of PS1, is required for PS1 endoproteolysis and γ-secretase activity [70]. Recently, Yamasaki *et al*. reported that the PS1 L383F mutant (affecting substrate identification as described below) was endoproteolytically processed and allowed maturation of NCT, suggesting that this residue does not play an important role in the assembly of the complex [71].

###### TMD8

Shiraishi *et al*. reconstituted recently γ-secretase activity by co-expressing C-terminally truncated PS1 (lacking γ-secretase activity), the corresponding PS1 C-terminal deleted fragment and PS cofactors NCT, APH-1 and PEN-2 in PS1/PS2 double deficient MEFs [72]. In this study, the expression of PS1-C68 (PS1 C-terminal 68 residues containing TMD8 and TMD9) rescued a defect in the γ-secretase activity of PS1∆C66 (PS1 lacking the C-terminal 66 residues containing TMD8 and TMD9). Similarly, the expression of PS1-C37 (PS1 C-terminal 37 residues containing TMD9) rescued a defect in the γ-secretase activity of PS1∆C37 (PS1 lacking the C-terminal 37 residues containing TMD9). However, the expression of PS1-C37 (containing TMD9) failed to rescue a defect in the γ-secretase activity and assembly of PS1∆C66 (lacking TMD8 and TMD9), demonstrating that TMD8 is critical for both the assembly and the activity of γ-secretase [72].

###### “PAL” Motif (TMD9)

Wang and al. recently investigated the molecular mechanism by which the essential proline-alanine-leucine (PAL) motif in PS1 (residues 433-435 in human PS1) contributes to γ-secretase activity [73, 74]. Single point mutations to this motif (P433L, A434D and L435R) completely abolish γ-secretase activity [73, 75]. Originally proposed to be important for the assembly and stabilization of the γ-secretase complex (a hypothesis that was not consistent with the fact that this motif is conserved in SPP, which unlike PS1, does not require cofactors for activity), Kaether *et al*. and Wang *et al*. later demonstrated that this motif was not involved in the γ-secretase complex formation [73]. Indeed, the P433L PS1 mutations did not affect γ-components stability, trafficking and incorporation into the active high molecular weight complex [74]. Nevertheless, mutations at this motif completely abolish binding to a transition-state analog inhibitor of γ-secretase, supporting the hypothesis that this motif, like the TMD1, is important for the proteolytic mechanism of γ-secretase [74]. Consistent with an enzymatic function of the PAL motif, the same sequence is also essential for SPP activity. Interestingly, the PAL sequence is located within the final TMD in the 9-TMD topological revised model, as expected for such a motif to be directly involved in an intramembrane-cleaving activity [74]. 

#### 2 Nicastrin: Stabilizer of the γ-Secretase Complex & Substrate Receptor

NCT was the second member of γ-secretase to be identified (year 2000), initially detected through a genetic screen in *C. elegans *[76] and isolated later biochemically from mammalian cells following partial affinity purification using a PS1-directed antibody [77]. Human NCT is a 709 amino acid residues long single-pass type I membrane protein (Fig. (**2**)) that possesses an N-terminal predicted signal peptide, a large N-terminal hydrophilic ectodomain with 16 potential glycosylation sites, one hydrophobic TMD and a short hydrophilic C-terminus of 20 amino-acid residues [77]. NCT exists in cells under three principal forms: (1) the unglycosylated, nascent protein (~70-80 kDa), (2) the “immature” N-linked isoform (iNCT; ~110 kDa) that is mainly located within the ER (endoglycosidase-H-sensitive glycosylation pattern) and is not associated with active γ-secretase, and (3) the “mature” N-linked isoform (mNCT; ~130-150 kDa) that is formed after entering the Golgi apparatus (complex glycosylation pattern) and trans-Golgi network (sialylation) [78-82] and is found only in the active complex.

##### Domains of Nicastrin Critical for the Assembly and/or the Activity of the γ-Secretase Complex

Research has focused on the domains of PS that are critical for γ-secretase activity (see above), but less is known about the domains of NCT that are essential for the assembly and/or the activity of γ-secretase. Fibroblast cells isolated from two independently generated NCT KO mouse strains have been used to investigate the functions of NCT within the γ-secretase complex [83-85]. Reduced matured PS fragments (PS-NTF & PS-CTF) have been observed in these three different studies. Moreover, down-regulation of NCT in different cell lines by small interfering RNA (siRNA) revealed a reduction of PS fragments [17, 84, 86-88]. When taken together, those data support an essential role for NCT in maintaining the stability of the complex. 

###### NCT Ectodomain Including the Conserved “DYIGS” Motif

Three domains (residues 306-360, 419-458, and 625-662 in human NCT) with significant sequence conservation have been identified in orthologous NCT genes (from different species) including Human, Mouse, *Drosophila melanogaster* and *C. elegans* [77]. Within the first conserved domain, all four proteins contained the motif DYIGS (residues 336-340 in human NCT) which is apparently a PS binding domain [77]. Indeed, deletion of the DYIGS motif caused a significant reduction in the NCT/PS1 interaction, as well as a reduction in both Aβ42 and Aβ40 secretion in HEK293 cells stably overexpressing APP with Swedish mutation and the mutant NCTs [77, 89]. Murphy *et al*. confirmed that mutations in DYIGS motif (amino-acid deletions of residues 312-340 or 312-369) resulted in the loss of function phenotype with respect to γ-secretase processing of APP-based substrates [90]. Intriguingly, mutants of the DYIGS motif (DYIGS to AAIGS [77] or D336A/Y337A [89]) caused a significant increase in Aβ secretion [77, 89]. In an attempt to identify the functionally important domains of NCT, Shirotani *et al*. generated a set of deletions and point mutations within the NCT ectodomain and probed the corresponding cDNAs for their faculty to restore γ-secretase assembly and activity in a HEK293 cell line in which endogenous NCT expression was stably knocked-down by RNAi [86, 87]. All investigated deletions within the ectodomain (including the DYIGS motif) as well as mutations in (G339A) or close to (E333Q) the DYIGS motif failed to restore those functions, suggesting an important role of the entire ectodomain in γ-secretase assembly and activity [86, 87]. Two conserved residues (S632 and W648) in the juxtamembrane region of the NCT ectodomain have also been identified as essential residues for γ-secretase complex formation and activity [91]. In contrast, the cytoplasmic tail of NCT was found to be dispensable for γ-secretase complex assembly and activity [92]. 

The loss of function phenotype observed in most of the NCT mutants of the DYIGS motif described above can be explained by the recent finding that NCT functions as a γ-secretase-substrate receptor that recognizes the short N-terminal stubs generated by ectodomain shedding of type-I membrane proteins [19]. Indeed, Shah and colleagues found that the NCT ectodomain (but not the cytoplasmic and TMD regions) physically interacts with APP- and Notch-based substrates in co-immunoprecipitation experiments performed with Triton X-100 membrane extracts from baculovirus infected Sf9 cells expressing different NCT constructs plus APP-C99 or N100, a Notch-based fragment resulting form TACE cleavage [19]. Next, NCT harboring a deletion within its ectodomain (NCT∆312-340 including the DYIGS motif) or a mutation close to the DYIGS motif (NCT-E333A) failed to restore γ-secretase activity in NCT-/- fibroblasts and failed to interact physically with APP-C99/C83 in the same cells [19]. Supporting this observation, the NCT ectodomain including the residues 306-360 (the most conserved sequence amongst NCT orthologs) shows sequence similarity to a peptidase family including aminopeptidases characterized by a glutamate residue in the active site that interacts directly with the substrate [93-95]. Based on this observation and using known aminopeptidase structures as a model, Shah *et al*. postulate that the highly conserved glutamate residue at position 333 of NCT is positioned in an analogous substrate-binding pocket within γ-secretase [19]. When taken together, these data demonstrate that a substrate binding site that is evolutionarily derived from a peptidase and including the DYIGS motif as well as the proximal glutamate residue at position 333 resides in the ectodomain of NCT.

###### The TMD of NCT

The TMD of NCT is required for the interaction with γ-secretase complex components and for γ-secretase activity [92, 96]. To avoid the misfolding phenotype often associated with introducing deletions or mutations in glycosylated and disulfide bonded proteins [97] (such as NCT), Morais *et al*. developed a strategy based on the finding that *C. elegans* NCT failed to associate with human γ-secretase components [96]. They produced chimeras between human NCT and *C. elegans* NCT and examined their ability to interact with human γ-secretase components as judged by co-immunoprecipitation. The authors found that the TMD of NCT as well as regions immediately adjacent to it (amino-acid residues 620-709 of hNCT) play important roles in mediating protein:protein interactions between γ-secretase components as well as γ-secretase activity [96]. A sequential deletion analysis further indicated that the N-terminal one-third of the NCT TMD is sufficient for the assembly of NCT into the γ-secretase complex but also that the remaining C-terminal portion contributes to some extend to γ-secretase formation [92]. Moreover, a helical wheel projection of the NCT TMD revealed a hydrophilic patch which seems to be essential for the assembly within a functional γ-secretase complex [92]. When taken together, these data demonstrate that the TMD of NCT is important for both the assembly and the activity of γ-secretase.

#### 3 Anterior Pharynx Defective (APH-1)

In 2002, two independent but very similar genetic screens in *C. elegans* have revealed two additional PS and NCT cofactors, APH-1 and PEN-2 (Fig. (**2**)) required for processing of PS into PS-NTF and PS-CTF heterodimers and for γ-secretase activity [98, 99]. In *C.* *elegans*, the Notch signaling pathway is required for the development of the anterior half of the pharynx, and embryos defective in this requirement have an Aph (anterior pharynx) defective phenotype [76]. Worms that are defective in both PS genes (hop-1 and sel-12) display all the hallmarks of Notch signaling defects including maternal-effect embryonic lethality and germline sterility. In this screen, Francis *et al*. mutagenized single sel-12 mutants or single hop-1 mutants (which are partially compromised for PS function, fully viable and fertile), and screened for mutants that display the Notch-like sterility phenotype seen in the double hop-1 and sel-12 mutants [99]. Two homologues of Aph-1 were identified, Aph-1a (mapped to chromosome I) and Aph-1b (mapped to chromosome 15) that at the genetic level interact with hop-1 and sel-12. At the protein levels, APH-1a exists in two C-terminal splice forms, the 247-amino acid splice variant APH-1aS and the 265-amino acid splice variant APH-1aL which are identical except at the C-terminus [99-101]. Human APH-1a and the 257-amino acid long APH-1b share 50% sequence homologies [100, 101]. Whereas APH-1a is the principal APH-1 isoform present in γ-secretase complexes [102-104], the three different human APH-1 variants are able to form distinct active γ-secretase complexes [105, 106]. No penetrant mutations in the genes encoding Aph-1a and Aph-1b have been linked with the rare cases of familial AD [107].

##### Transmembrane Topology of APH-1

Hydropathy plot analysis by the Kyte-Doolittle method shows that APH-1 proteins are largely hydrophobic, with a common pattern of seven hydrophobic regions that are predicted to be membrane-spanning regions [98, 108]. The orientation with regard to luminal and cytosolic compartments has been investigated by Fortna *et al*. and using different experimental approaches [108]. Immunofluorescence microscopy performed under various and selective permeabilization conditions of the plasma membrane strongly supports a model in which the C-terminus of APH-1 faces the cytosolic space [108]. Furthermore, the method of glycosylation-scanning mutagenesis (proteins containing this motif will be glycosylated only if the sequence is located within an ER luminally exposed region) revealed that the N-terminal domain as well as the predicted loops 2, 4, and 6 were glycosylated, but not loops 1, 3, and 5. When taken together, these experiments offer a seven TMD model for the topology of APH-1 in which the N-terminus faces the ER lumen and the C-terminus resides in the cytosolic space (Fig. (**2**); [108]).

##### Domains of APH-1 Critical for the Assembly and/or the Activity of the γ-Secretase Complex

###### The “GXXXG” Motif: An Important Determinant in Transmembrane Helix:Helix Interactions

The conserved Gly122, Gly126 and Gly130 in the APH-1 family of proteins (TMD 4 of mammalian APH-1aL) have been identified as critical for γ-secretase assembly and activity [109-111]. These glycine residues are part of the G*XXX*G motif (where X represents any amino acid), generally accepted as a major motif in transmembrane helix:helix protein interactions [112, 113]. Consistent with this function, mutations of Gly122, Gly126 and Gly130 in the G*XXX*G motif prevent the stable association of APH-1aL with PS and NCT, suggesting that APH-1 is a major docking site for the assembly and consequently for the activation of γ-secretase [109]. Similar results were found in Drosophila S2 cells by using an RNAi-based complementation assay [110]. Further supporting the function of APH-1, Saito and colleagues recently identified a novel 216 amino acid splice variant of human APH-1b which lacks the normal exon 4 (APH-1b∆4) and consequently the G*XXX*G motif described above [114]. Consistent with the function of the G*XXX*G motif, APH-1b∆4 was less stable than APH-1b and APH-1b∆4 was stabilized by NCT [114]. When taken together, the results described above indicate that the G*XXX*G transmembrane motif is essential for the stability of the APH-1 protein, the assembly of the γ-secretase complex as well as the activity of γ-secretase.

#### 4 Presenilin Enhancer-2 (PEN-2)

PEN-2 was detected in 2002 through two independent but very similar genetic screens in *C. elegans* [98, 99]. The corresponding human gene is located on chromosome 19 and encodes a 101 amino acid residues long protein. Downregulation of PEN-2 expression in different cell lines by small interfering RNAs (siRNA) abolishes the endoproteolysis of PS (associated with a significant accumulation of full-length PS) and γ-secretase activity, whereas overexpression of PEN-2 promotes the cleavage of PS, indicating an important role for PEN-2 in PS-NTF and-CTF heterodimer formation [17, 115-117]. Recently, Pen-2 exons and nearby intronic regions were analyzed for the presence of mutations and/or polymorphisms in an Italian population sample of 140 familial AD patients, 256 sporadic AD patients, and 253 cognitively normal elderly people as control group [118]. Scanning the gene revealed the PEN-2 D90N very rare missense mutation in a patient with familial AD – however, the pathogenic role of this mutation is not clear as preliminary data point to no influence of this mutation on APP processing and Aβ42 production [118]. 

##### Transmembrane Topology of PEN-2

The PEN-2 proteins identified in different species including human, mouse, Drosophila, *C.* *elegans *and zebrafish show high sequence similarity throughout their length, contain two predicted TMDs, and lack a signal peptide as well as any previously described and characterized conserved protein motifs [99]. The membrane topology of human PEN-2 has been investigated by Crystal *et al*. using three different and complementary biochemical approaches: glycosylation-scanning mutagenesis (used by Fortna *et al*. to support a model for the membrane topology of APH-1, see above), immunofluorescence microscopy as well as a protease protection assay using selective permeabilization conditions of the plasma membrane [119]. Based on their results, the authors came to a conclusion that PEN-2 spans the membrane twice (thus confirming the two predicted TMDs), with the N- and C-termini facing the ER lumen and the hydrophilic loop facing the cytosolic space (Fig. (**2**); [119]).

##### Domains of PEN-2 Critical for the Assembly and/or the Activity of the γ-Secretase Complex

###### The TMD1 of PEN-2

The TMD1 of PEN-2 is critical for PS endoproteolysis and γ-secretase activity. The “Swap method” (replacement of PEN-2 TMDs with TMDs of sterol regulatory element binding protein 1 (SREBP-1)) was used for the analysis of the individual roles of PEN-2 TMDs for PS endoproteolysis and γ-secretase activity [120]. The replacement of TMD2 of PEN-2 with the TMD1 from SREBP-1 was fully functional whereas the replacement of TMD1 of PEN-2 with the TMD2 from SREBP-1 failed to trigger PS endoproteolysis [120]. This loss of function was further rescued by the replacement of the two-thirds of the SREBP-1 TMD2 with the two-thirds of the TMD1 of PEN-2 [120]. When taken together, these data suggest that the two-thirds of the TMD1 of PEN-2 are functionally important (in contrast to the TMD2 of PEN-2) for PS endoproteolysis and γ-secretase activity.

###### The C-Terminal Highly Conserved DYLSF Motif of PEN-2

The C-terminal highly conserved DYLSF motif of PEN-2 is required for the stabilization of PS1 fragments (NTF & CTF). To define the PEN-2 functional domains, Prokop *et al*. initiated a structure-function analysis of PEN-2 using a HEK293 cell line in which PEN-2 expression is stably knocked down by RNAi – loss of PEN-2 function (associated with reduced PS fragments and accumulation of PS-holoprotein) can thus be assayed by functional complementation [121]. Steric alterations of the C-terminus of PEN-2 by the addition of a C-terminal myc-hexahistidine tag or by a C-terminal truncation of 17 amino-acid residues did not allow the rescue of the defect in γ-secretase maturation, suggesting that the C-terminal domain of PEN-2 is a functionally critical domain [121]. In addition, alteration of the C-terminus of PEN-2 caused a selective instability of the PS1 heterodimers that underwent proteasomal degradation, supporting a critical role of the C-terminus of PEN-2 in the stabilization of the PS fragments and consequently in the assembly and activation of the complex [121]. The hydrophilic C-terminus and more specifically the amino acid residues 90-101 of PEN-2 are conserved during evolution with the possibility to be functionally important [122]. Indeed, intensive mutation analysis within this domain revealed that the C-terminus of PEN-2 and more specifically the highly conserved DYLSF motif at residues 90-94 is important for the physical interaction of PEN-2 to PS (thus stabilizing the PS fragments) and the maturation and activation of γ-secretase [122]. Consistent with these findings, two deletions in the N-terminal region (∆3-9 and ∆10-16), two in the loop between TMD1 and TMD2 (∆40-46 and ∆52-60) and two in the C-terminal region (∆85-92 and ∆93-100) had no or very little effects on the endoproteolysis of PS1 and γ-secretase activity in contrast to the deletion of the whole C-terminal region (∆85-101, including the conserved DYLSF motif at residues 90-94), further supporting the critical function of the C-terminal hydrophilic region of PEN-2 for γ-secretase activity [120, 123]. The overall DYLSF motif is the critical functional determinant of PEN-2 as individual D90A and F94A mutations are functionally tolerated [123].

### Assembly and Activation in the Secretory Pathway of the γ-Secretase Complex (Fig. (3))

The glycosylation pattern of NCT and its conversion from an immature to a mature form within the secretory pathway has been extensively used to investigate the assembly and trafficking of the γ-secretase complex in different cell lines (including CHO, HEK293, N2a, mouse embryonic fibroblasts and neurons). Basically, newly synthesized mammalian proteins are co-translationally modified in the ER by the addition of 14 oligosaccharide units, including 9 mannose residues, 3 glucose residues and 2 *N*-acetylglucosamine residues (= N-linked glycosylation) [124]. In the medial/trans-Golgi compartments, 6 of the mannose residues can be replaced with complex sugars including N-acetylglucosamine, galactose, sialic acid, fucose or chondroitin sulfate glycosaminoglycans [124]. The nature of such post-translational modifications of NCT has been addressed by taking advantage of the differential susceptibility of glycoproteins to enzymes targeting specific post-translational modifications. First, digestion of iNCT and mNCT with PNGase F (peptide-N-glucosidase F), an enzyme that cleaves all N-linked (but not O-linked) glycans regardless of complexity decreases the size of NCT to a size corresponding to the unglycosylated, nascent 70-80 kDa protein [80-82, 125]. Combined with the observation that (1) treatment with enzymes that cleave all O-linked (but not N-linked) glycans did not affect SDS-PAGE mobility of iNCT and mNCT [80-82], and (2) lectins specific for N- glycans, in contrast to lectins specific for O-glycans, do bind to iNCT and mNCT [81, 82], these results demonstrate that NCT is modified by N- but no O-linked glycosylation. Endoglycosidase-H (Endo-ß-N-acetylglucosaminidase H, Endo-H) is a highly specific endoglycosidase cleaving asparagine-linked mannose rich oligosaccharides, but not highly processed complex oligosaccharides from glycoproteins. Thus, glycoproteins within the ER and cis-Golgi compartments are Endo-H sensitive whereas complex glycoproteins within the medial- and trans-Golgi and later compartments (endosomes and plasma membrane) are Endo-H resistant. Consequently, Endo-H was used to differentiate the two forms of NCT, iNCT and mNCT [80-82, 125]. Collectively, treatments with Endo-H revealed that a majority of mNCT is Endo-H resistant [80-82, 125] whereas iNCT is Endo-H sensitive [80-82, 125], demonstrating that iNCT and mNCT are located in different cell compartments: ER and cis-Golgi for iNCT and medial-Golgi, trans-Golgi and later compartments for mNCT. Velocity sedimentation and subcellular fractionation of cells in iodixanol gradient further support the subcellular localization of iNCT and mNCT. Indeed, iNCT was primarily found in ER-enriched fractions whereas mNCT was found in Golgi-enriched fractions [80, 81].

#### 1 The iNCT/APH-1 Subcomplex: Early Formation in the Endoplasmic Reticulum

We and others proposed that the assembly of the γ-secretase complex involves the early formation in the ER of an intermediate complex (= subcomplex) of APH-1 and the “immature” N-linked isoform of NCT (iNCT; 110 kDa) that is not associated with active γ-secretase [48, 87, 117]. Indeed, the analysis under non-denaturing conditions using native gel electrophoresis (BN-PAGE) of γ-secretase components solubilized from CHO and HEK293 cells revealed a subcomplex of 140 kDa which was immunoreactive for NCT and APH-1 but not for PS-heterodimers or PEN-2 [117]. The size of this subcomplex is consistent with the predicted size of iNCT (110 KDa) plus APH-1 (20 kDa). Further supporting a specific interaction between iNCT and APH-1, co-immunoprecipitations experiments (co-IP) in multiple cell lines using antibodies against ectopically expressed as well as endogenous APH-1 revealed a selective association between those two proteins [117]. In working towards purification of the γ-secretase complex, we expanded our analysis of detergents that keep the complex together and maintain activity [48]. During this process, we identified one detergent (dodecyl-β-D maltoside, DDM) that, in contrast to 1% digitonin, dissociated endogenous and overexpressed active γ-secretase complexes into two major partial inactive complexes, one containing PS1-NTF and PEN-2 and the other containing mNCT and APH-1 (as characterized by Blue native BN-PAGE combined with a second dimension using SDS-PAGE in order to break apart these partial complexes) [48]. The DDM-resistant interactions between PS1-NTF and PEN-2 and between mNCT and APH-1 likely reflect the physical interactions that exist in the active γ-secretase complex. Assuming that the physical interactions revealed in the DDM-dependent dissociated complexes reflect the order of assembly of the complex (strong interactions that do not require additional γ-secretase members would correspond to direct and specific interactions), our findings would suggest that the DDM-resistant mNCT:APH-1 complex reflects the iNCT:APH-1 subcomplex generated early during the assembly of γ-secretase. In another study, three different mutations within conserved amino acid residues within the ectodomain of NCT caused the accumulation (in HEK293 cells) of iNCT variants which selectively bound to APH-1, independently to PS and PEN-2 [87]. Whether APH-1 stabilizes the unglycosylated, nascent protein (80 kDa), and allows its glycosylation in the ER or whether the post-translational modification of the nascent NCT to iNCT is required for its interaction with APH-1 remains unknown. When taken together, these data demonstrate that APH-1 and iNCT form a stable intermediate in the ER that would likely serve as a scaffold for the binding of PS and/or PEN-2 during the assembly of the active γ-secretase complex.

#### 2 The iNCT/APH-1/PS-Holoprotein Subcomplex

Evidence exists that PS holoprotein then binds to the iNCT/APH-1 subcomplex to form a second intermediate, prior to the addition of PEN-2 and full complex maturation. First, PS is required for the trafficking of NCT to the Golgi apparatus [80, 82]. Indeed, the complete absence of PS in PS1-/- PS2-/- double knock out MEFs promotes the accumulation of an Endo-H sensitive iNCT [80, 82]. Immunocytochemical experiments performed on the same PS1-/- PS2-/- MEFs further revealed that the accumulated iNCT was largely located in the ER, suggesting that NCT needs to associate with PS to leave the ER [82]. Next, velocity method to fractionate subcellular organelles on continuous iodixanol gradients revealed a high degree of co-localization in CHO cells and WT MEFs of iNCT with PS1 holoprotein in ER enriched fractions and mNCT with PS heterodimers in the Golgi-enriched vesicle fractions [80, 81]. This supports the localization of immature PS (PS-holoprotein) and mature PS (PS heterodimers) to different cellular compartments and consequently an early association between iNCT and PS-holoprotein. In support of this, we found that DDM-dependent dissociation of endogenous and overexpressed active γ-secretase complexes generated a minor inactive complex containing mNCT, APH-1, and PS1-CTF as characterized by Blue native BN-PAGE combined with a second dimension using SDS-PAGE [48]. The NCT “DYIGS” motif (residues 336-340 of human NCT) has further been proposed to be a PS binding domain [77] whereas the APH-1 “G*XXX*G” motif has been proposed to be both a PS and/or NCT binding domain [109-111], thus supporting possible physical interactions between NCT and PS, and between APH-1 and PS and/or NCT, respectively. Finally, knock-down experiments using siRNAs directed against APH-1 and NCT reduced the formation of PS heterodimers without the stable accumulation of PS-holoprotein observed in cells with down-regulated PEN-2. This observation suggests that (1) APH-1 and NCT could be involved in the stabilization of PS-holoprotein and (2) that APH-1, NCT and PS-holoprotein stabilize each other, independently of PEN-2. When taken together, these results support the existence of a subcomplex containing iNCT/APH-1/PS-holoprotein that has never been detected under physiological conditions, probably due to the very short half-life (1-2 hours) of PS-holoproteins when compared to that for PS-heterodimers (> 24 hours) [53] and their rapid endoproteolysis into PS-NTF and PS-CTF fragments. 

#### 3 The PEN-2 Dependent Maturation of PS Holoprotein

The endoproteolytic activity that cleaves PS-holoprotein into PS-NTF and PS-CTF fragments has the characteristics of an aspartyl protease and most γ-secretase inhibitors block PS endoproteolysis, supporting the hypothesis that PS endoproteolysis is an autocatalytic event [126, 127]. A model for this autocatalytic endoproteolytic processing of PS has been recently reported in which the same γ-secretase active site within PS (D257 and D385 of human PS1) that cleaves APP- or Notch-based substrates also cleaves and activates PS itself [56]. In this model, the hydrophobic domain that contains the site of endoproteolysis is membrane-embedded (in a close proximity to TMD 6 and TMD 7), thus occluding the active site in the conformation adapted by a PS holoprotein – a hypothesis that is supported by the observation that PS holoprotein is not associated with active γ-secretase, except for the FAD PS1∆E9 variant which lacks the PS endoproteolytic site but triggers γ-secretase activity. Next, the association of PS-holoprotein with γ-secretase would likely trigger a conformational change allowing the PS active site to cleave the E9 hydrophobic domain between the residues M292 and V293, resulting in the withdrawal of the hydrophobic domain around the active site residues that consequently becomes accessible for cleavage of γ-secretase substrates. Consistent with this model, evidence suggests that the association of PEN-2 to the trimeric iNCT/APH-1/PS-holoprotein subcomplex triggers the conformational change (in PS-holoprotein?) promoting the endoproteolytic cleavage of PS-holoprotein and consequently the activation of γ-secretase (depicted in Fig. (**3**)). First, knock-down experiments using siRNAs directed against PEN-2 lead (1) to reduced PS heterodimers, mNCT, active and stable γ-secretase complex, and (2) to the stable accumulation of PS-holoprotein [17, 115-117]. The latter observation is in contrast to knock-down experiments using siRNAs directed against APH-1 and NCT which trigger reduced formation of PS heterodimers without the stable accumulation of PS-holoprotein observed in cells with down-regulated PEN-2 [17, 88, 116, 117]. This supports a critical function of PEN-2 in the endoproteolysis of PS-holoprotein and the maturation of the complex. Moreover, the accumulation of iNCT in cells with down-regulated PEN-2 supports a direct interaction of PEN-2 with the trimeric iNCT/APH-1/PS-holoprotein subcomplex in the ER/cis-Golgi compartments [116]. Next, the overexpression of PEN-2 in different cell lines facilitates PS-holoprotein endoproteolysis [17, 116]. Also, PEN-2 preferentially binds both PS-holoprotein and mNCT in our experiments, suggesting that this component binds to the complex at a later stage of assembly and plays a critical role in the endoproteolysis of PS [117]. In support to this, we found that in cells stably overexpressing only PS1 and Flag-PEN-2, antibodies directed to the very C-terminus of PS1-CTF (this domain is inaccessible to those antibodies when folded into the active complex) could co-IP PEN-2 and anti-Flag antibodies brought down full-length PS1 [48]. This observation demonstrates that PEN-2 can associate with full-length PS1 in a manner independent of NCT, APH-1, and PS1-NTF, suggesting that PEN-2 and PS1-holoprotein are capable of interacting physically with each other. The detergent-dependent dissociation of active γ-secretase further revealed an interaction between PEN-2 and PS1-CTF [48]. Finally, the preferential association of PEN-2 with mNCT supports a critical role for PEN-2 in a late stage during the assembly of γ-secretase. Indeed, we recently developed protocols for the high-grade purification of proteolytically active γ-secretase [21, 128]. As a last step of purification, we developed an M2 anti-Flag antibody immuno-affinity procedure, a separation based on the presence of Flag-PEN-2 in the active complex [21, 128]. We found that mNCT and PS1 heterodimers (PS1-NTF & PS1-CTF) were enriched after the M2 anti-Flag antibody immuno-affinity purification of Flag-PEN-2, further supporting several reports that only mature NCT and mature PS heterodimers are associated with the active γ-secretase complex [80, 81, 129].

#### 4 The mNCT/APH-1/PS-NTF/PS-CTF/PEN-2 Active Complex

When taken together, the results described above strongly support a model in which PEN-2 is added to the trimeric iNCT/APH-1/PS-holoprotein subcomplex in the ER/cis-Golgi compartments and promotes the trafficking and activation of γ-secretase. The fully matured and active γ-secretase (mNCT/APH-1/PS-NTF/PS-CTF/PEN-2) has been reported to be mainly localized in cell compartments including the Golgi [130-132], trans-Golgi network (TGN) [131, 133], endosomes [133-135], lysosomes [136-138], and plasma membrane [134, 135, 139-143]. 

### Three-Dimensional Structure of the γ-Secretase Complex

γ-Secretase is the founding member of an emerging class of *i*ntramembrane-*cl*eav*i*ng *p*roteases (I-CliPs) that includes site 2 protease (S2P), Rhomboids and signal peptide peptidase (SPP) (for a review, see [144]). Like γ-secretase, S2P, Rhomboid, and SPP all hydrolyze their respective substrates within their TM regions, and the residues responsible for catalysis are either intramembranous or at the predicted membrane-cytosol interfaces of the proteases. Rhomboid and SPP function as a single component that can be overexpressed and purified from bacteria with catalytic activity [145, 146] and in amounts adequate for crystallization studies [26-28, 147]. In sharp contrast, γ-secretase is a multi-component protease with complex assembly, maturation (including N-linked glycosylation of NCT, a post-translational modification that does not occur in prokaryotic systems) and activation processes. It is thus likely that prokaryotic systems are not appropriate for the overexpression of physiological γ-secretase for crystallization studies. Indeed, γ-secretase has never been overexpressed in an active form in bacteria and the PS endoproteolytic event, which has been associated with the biological activity of γ-secretase, has never been reproduced in prokaryotic systems. However, proteolytically active γ-secretase has been reconstituted in *S. cerevisiae* which lacks this enzyme [20]. Nevertheless, only a small fraction of the overexpressed PS holoprotein was endoproteolysed into active NTF- and CTF fragments [20], suggesting that the yeast system is currently unsuitable for the production of large quantities of purified complexes for structural studies. Functional γ-secretase has also been reconstituted in the baculovirus/insect cell system by co-infecting all four components (His/Flag-PS1, NCT-V5/His, APH-1-myc/His, and PEN-2) into *Spodoptera frugiperda* Sf9 cells [148]. This system was used to develop a three-step procedure (anti-FLAG immunoaffinity chromatography, size exclusion chromatography and Ni-NTA affinity chromatography) for the purification of active γ-secretase [149]. To assess the 3-dimensional structure of γ-secretase, we developed a specific and reproducible protocol for the high-grade purification of intact, proteolytically active γ-secretase complexes from Chinese hamster ovary (CHO) cells that stably overexpress human PS1, APH-1-HA and Flag-PEN-2 in the presence of high levels of endogenous NCT (designated γ-30 cells) [21]. 

#### 1 A Water-Containing Chamber in the Catalytic Core of γ-Secretase Provides the Hydrophilic Environment Required for Proteolysis

Electron microscopy (EM) and single particle image analysis of γ-secretase purified from the γ-30 CHO cells were used to determine the first three-dimensional structure of γ-secretase at a resolution of 15 A° [22]. First, EM revealed γ-secretase particles that measured 80-100 Å in diameter, consistent with particles of ~300 kDa in mass, a size that has been reported using biochemical sizing methods, including BN-PAGE [21, 48, 117]. Next, a total of 12,000 individual particle images were used to generate the refined 3D reconstruction [22]. Purified, active γ-secretase was found to have an elongated globular structure, with the largest dimension being ~120Å and the other two dimensions perpendicular to the long axis being about 70-80 Å each [22]. The most dramatic feature of the structure was the presence of a low-density central (and possibly water-containing) intramembrane chamber with a diameter of 20-40 Å (see the cut-open view displayed in Fig. (**4**); [22]). In a different but very similar study, EM and analysis of 2341 negatively stained γ-secretase particles purified from the baculovirus/insect cell system resulted in the 3D-representation of γ-secretase at a resolution of 48 Å [149]. Supporting the possible existence of a water-containing chamber, the reconstruction of the γ-secretase complex purified from the baculovirus/insect cell system revealed an elongated structure with a large pore in the center [149]. Experimental and biochemical evidence for the existence of a water-filled cavity in the intramembranous part of γ-secretase has been provided by using a cysteine-scanning mutagenesis approach [150, 151]. The principle involves substitution of amino-acid residues of interest with cysteine and the use of membrane-permeable or –impermeable sulfhydryl-directed reagents. Cysteines embedded in the membrane, unless exposed to a water-containing cavity, are not reactive with such reagents. Taking advantage of the unique properties of cysteine-scanning mutagenesis, Tolia *et al*. found that cysteines introduced at several positions in TMD 6 and TMD7 of PS1 (including the catalytic Asp-385) were accessible to N-[6-(biotinamido)hexyl]– 3’-(2’-pyridyldithio)-propionamide (EZ-link biotin-HPDP), a sulfhydryl-specific reagent, implying that these residues have access to water within the lipid bilayer [150]. The authors also propose that Y389 of PS1 is involved in the catalytic mechanism through the formation of a hydrogen bond with the side chain of D385 and found that the membrane-permeable and biotinylated sulfhydryl-specific cross-linker did not label D257C (inaccessible to water?), whereas a homobifunctional sulfhydryl-specific cross-linker did target both D257C and D385C (both accessible to water) [150]. A model is proposed to explain this apparent discrepancy, based on functionally distinct complexes. This model suggests that D257 of PS1 remains inaccessible to water in inactive γ-secretase, but does get access to water during hydrolysis in active γ-secretase, possibly upon conformational modulations within TMD6 initiated after binding of the substrates. In a similar study, Sato *et al*. further demonstrated that TMD6 and TMD7 of PS1 contribute to the formation of a hydrophilic pore within the membrane [151]. When taken together, these results provide biochemical evidence for the existence of a water-filled cavity in the catalytic core of γ-secretase. While the requirement for water molecules to accomplish peptide bond hydrolysis is expected, it represents the first experimental evidence that the catalytic aspartic residues are both accessible to water and face each other with a maximal distance of 5.2 Å.

#### 2 A Model for the Intramembrane Processing of Membrane Proteins by γ-Secretase

Our structural analysis of the purified γ-secretase complex further revealed an extracellular density at the top of the map which can be attributed to a part of the large (669 amino acid) ectodomain of NCT, as it comprises a long extracellular polypeptide (~70 kDa) onto which N-linked oligosaccharide groups are attached (Fig. (**4**); [22]). Moreover, two additional openings to the interior chamber were observed: one of ~20 Å size (designated H1) facing up towards the exterior of the cell, and the other about 10 Å in size (designated H2) facing down towards the cytoplasm (Fig. (**4**); [22]). Finally, two weak density regions (asterisks in Fig. (**4**)) were also observed on the attributed intramembrane surface [22]. 

When taken together, this 3D structure of γ-secretase provides plausible explanations for certain long-standing questions. Indeed, the most enigmatic feature of γ-secretase and other intramembrane proteases is their location within the hydrophobic environment of the membrane and yet their requirement for water molecules to accomplish peptide bond hydrolysis. How γ-secretase releases its two cleavage products into distinct subcellular compartments has also been mysterious. The 3D structure of γ-secretase and the structural details described above offer a model for the intramembrane processing of membrane proteins as depicted in Fig. (**5A**). In this model, the observed interior chamber and apical and basal pores would allow the entry of water molecules and their sequestration from the lipid surround by the proteinaceous microenvironment provided by the many transmembrane domains. The two thin-density regions in the transmembrane portion (labeled with stars in Fig. (**4**)) may represent sites which could be transiently opened up to enable entrance of the α -helically conformed substrates, i.e., the TMDs of various single transmembrane proteins. After a substrate (e.g., C99 of APP or the analogous N∆E fragment of Notch) enters the proteinaceous central chamber and undergoes hydrolysis by the 2-aspartate-containing catalytic site in PS, the cleavage product that includes the extracellular half of the substrate’s TMD (e.g., Aβ or Np3) could be released through the H1 channel to the extracellular surface, and the product that includes the cytoplasmic half of the substrate’s TMD plus the intracellular region (e.g., AICD or NICD) could be released through the H2 channel to the cytoplasm (Fig. (**5A**)).

#### 3 Crystal Structure of GlpG, an E. coli Intramembrane Protease of the Rhomboid Family: A Model for γ-Secretase?

Despite the recent progress, the low-resolution 3D structures of γ-secretase failed to answer some important questions. First, the stoichiometry and spatial organization of the complex are still under debate, with the number of PS molecules at the catalytic site of the complex being controversial. Indeed, evidence suggests that two PS molecules (a dimer) are at the catalytic core of a single protease complex [152-156]. In sharp contrast to this hypothesis, co-IP and western blot methods concluded that the γ-secretase complex contains only one copy of each component, demonstrating that the stoichiometry of the active mNCT/APH-1/PS-NTF/PS-CTF/PEN-2 complex is 1:1:1:1:1 ([157] and Fraering *et al*., unpublished). Next, how the catalytic site of γ-secretase is organized remains unknown. There is little ambiguity about the peptide bond scission being carried out by two aspartates in PS, but the locations of the active site and the oxyanion-binding site (that stabilizes the negative charge developed on the carbonyl oxygen of the scissile bond during catalysis) are not yet defined. Moreover, the molecular mechanisms explaining the access of the substrates to the catalytic site remain largely unknown. How is this access regulated? Do protease plasticity and substrate binding support a gate-opening mechanism as described for the GlpG rhomboid protease (four crystal structures ranging in resolution from 2.1 to 2.6 Å have been obtained almost simultaneously by three different laboratories [26-28, 147])? The 2.1 Å resolution crystal structure of a truncated but proteolytically active version of the *E. coli* GlpG rhomboid protease (named GlpG core domain) provided the first atomic-scale representation of an intramembrane protease [26]. While the requirement for water to accomplish peptide bond hydrolysis is expected, Wang *et al*. provide the first experimental evidence that the active site found in a central cavity that contains all the conserved polar residues (including the Ser-his catalytic dyad) accepts a number of water molecules [26]. Interestingly, a large V-shaped opening between two transmembrane helices (S1 and S3 in TMD1 and TMD3, respectively) and facing laterally towards the lipid is proposed to be the route (substrate docking site? Substrate binding site?) by which a substrate enters the active site [26]. Importantly, the crystal structure described in this report (and resolved in the absence of any substrate) shows that this lateral opening is blocked by a membrane-embedded loop structure (called L1). The authors postulate that L1 functions as a “lateral gate”, which may control substrate access to the active site. In contrast, the structures of GlpG reported by Wu *et al*. [27] and Ben-Shem *et al*. [28] lead to a structural model for gating in which TMD5 moves away from the catalytic site, carrying the L5 loop with it, to provide active site access for substrates passing between TMD5 and TMD2. By describing a new crystal structure of GlpG in a more open conformation, Wang and Ha demonstrated that L5 is indeed intrinsically flexible, consistent with the expectation that this loop can be displaced by the substrates in the “open-cap” state [147]. In a structure-function approach analyzing engineered GlpG variants, Baker *et al*. further implicated the helix in TMD5 as the lateral substrate gate [158]. Both studies further support the model first proposed by Wu *et al*. and Ben-Shem *et al*. in which the membrane substrates may enter the protease active site through the gap between TMD2 and TMD5 (Fig. (**5B**&**C**)). When taken together, the proposed gating mechanisms for the substrate access to the GlpG catalytic site are consistent with the model proposed by Brunkan *et al.* in which the membrane-embedded domain that contains the presenilin (PS) endoproteolysis site controls substrate access to the catalytic aspartates of γ-secretase by occluding the active site [56]. However, a major difference lies on the observation that γ-secretase activity depends on the processing of full-length PS (FL-PS) into PS-NTF and PS-CTF domains (resulting in the accessibility for the substrates to the active site), whereas the rhomboid L5 loop does not require this additional maturation step. 

Despite some important functional similarities, γ-secretase probably uses different and additional structural motifs to accomplish substrate selectivity and intramembrane proteolysis. For example, the NCT large extracellular domain has recently been shown to be essential for recognition of substrates by the γ-secretase complex [19]. The structure (and sequence) of GlpG does not display such a similar domain. Next, the 3D electron microscopic structure of the purified, proteolytically active γ-secretase, revealed that the NCT ectodomain covers the top of the large aqueous intramembrane chamber, suggesting a role as a type of flexible lid that could regulate (1) the entry of water molecules into the central chamber and/or (2) the exit of hydrophilic ectodomain products [22]. When taken together, it seems unlikely (but can not yet be excluded) that the short loop (L5) which tightly caps the GlpG active site from above fulfills similar functions. Co-crystallization of the GlpG core domain with a recombinant substrate would certainly provide a structural explanation for how a substrate can modulate the conformation of the proposed lateral gate, providing a good starting model for how substrates can be differentially handled in the catalytic site of γ-secretase. 

Finally, γ-secretase, site-2 protease (S2P), or the signal peptide peptidase (SPP) are unrelated to rhomboids by sequence or evolution - another major difference comes with the observation that γ-secretase only cleaves type-I membrane proteins whereas SPP only cleaves type-II membrane proteins. Awaited high-resolution structures of S2P, SPP and γ-secretase will solve the mystery of how they are similar and different.

## Figures and Tables

**Fig. (1):**
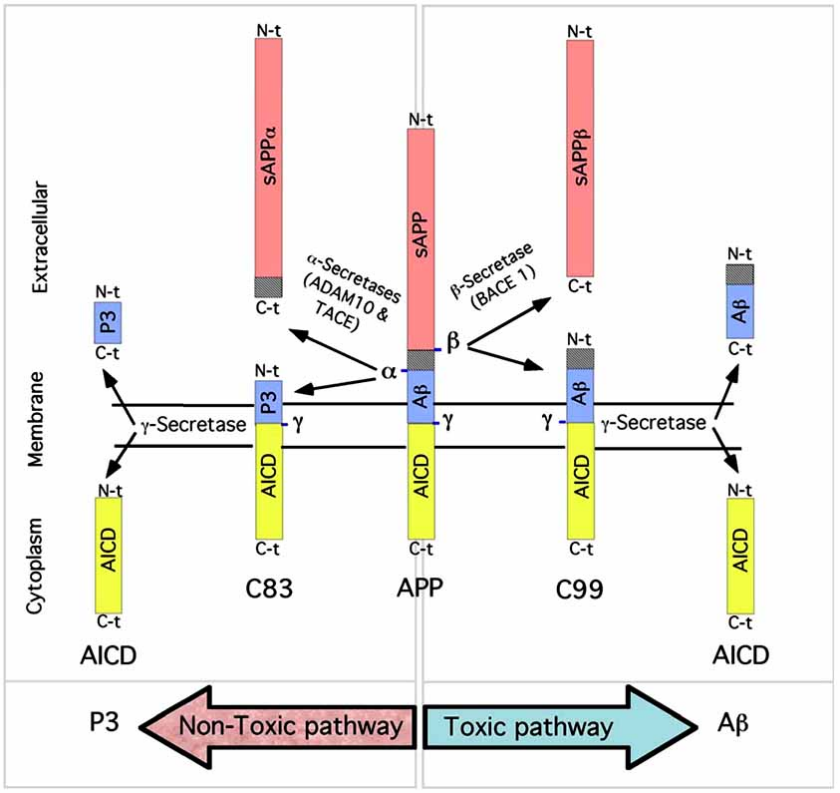
**APP processing and the production of the amyloid-β peptides.** APP is synthesized as a type I transmembrane protein which initially undergoes cleavage at either α - or β-secretase sites to release large ectodomains, designated sAPPα  or sAPPβ, respectively. Ectodomain shedding leaves membrane-embedded fragments, C99 (99-residue C-terminal APP fragment) or C83 (83-residue C-terminal APP fragment), which are substrates for γ-secretase cleavage. Proteolysis of C99 by γ-secretase promotes the extracellular release of the amyloidogenic fragments (Aβ, toxic pathway) whereas proteolysis of C83 by γ-secretase generates an N-terminally truncated and non- toxic Aβ protein of 3 kDa (P3, non-toxic pathway). Evidence suggests that the progressive accumulation/oligomerization/aggregation of Aβ in brain regions subserving memory and cognition is the primary cause of neurodegeneration in Alzheimer’s disease. Proteolysis of C99 and C83 by γ-secretase also releases the APP intracellular domain (AICD). Following association with the adaptor protein Fe65 and nuclear translocation, AICD is able to suppress the expression of the major apoE/lipoprotein receptor LRP1 by binding directly to its promoter [15]. Thus, APP processing is also involved in the regulation of brain apoE (a major genetic determinant of AD) and cholesterol metabolism.

**Fig. (2):**
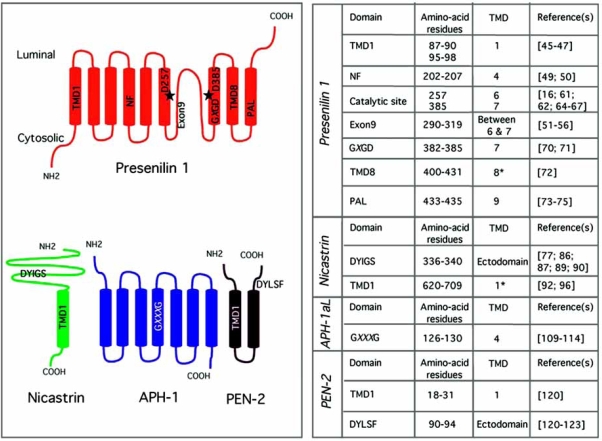
**The minimal set of components of the γ-secretase complex.**Topological models of human PS1, NCT, APH-1 and PEN-2 (left) showing the domains which have been experimentally determined to be critical for the assembly and/or activity of the γ-secretase complex (rigth). The transmembrane domains (TMDs) are shown as cylinders, stars represent the two catalytic aspartates (D257 and D385) in human PS1, and asterisks represent TMDs with N- or C-terminal regions immediately adjacent to them.

**Fig. (3):**
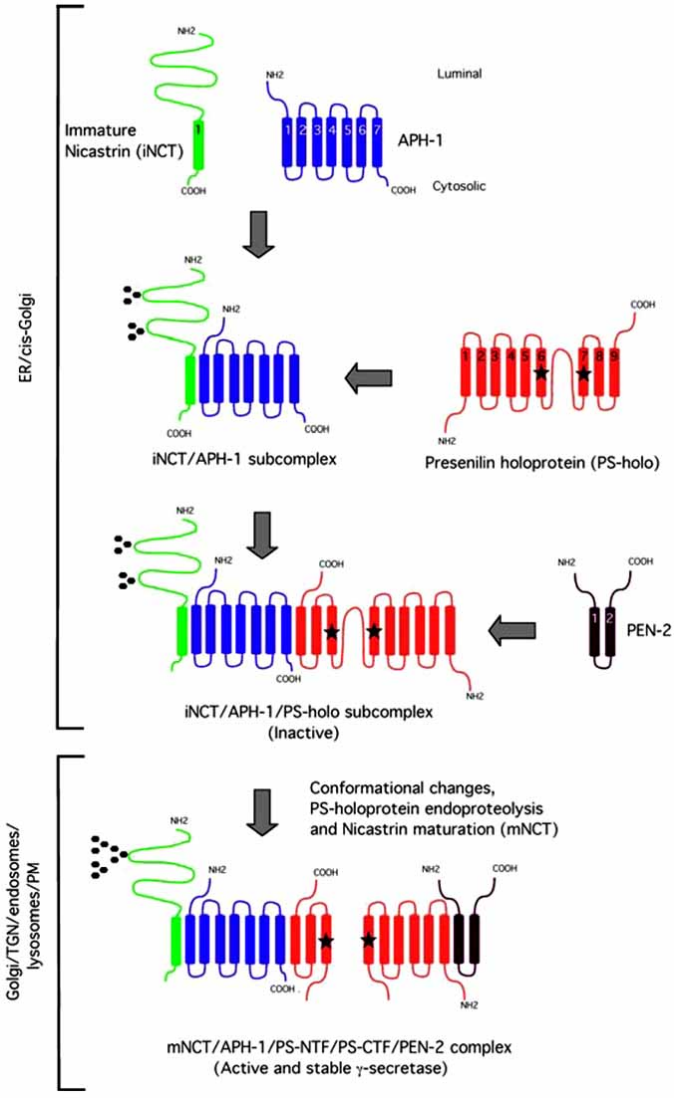
**Schematic representation of the assembly and activation in the secretory pathway of the γ-secretase complex.** The assembly of the γ-secretase complex involves the early formation in the ER of an intermediate subcomplex of APH-1 and the “immature” N-linked isoform of NCT (iNCT) that is not associated with active γ-secretase (= iNCT/APH-1 subcomplex). PS-holoprotein then binds to the iNCT/APH-1 subcomplex to form a second trimeric intermediate (= iNCT/APH-1/PS-holoprotein subcomplex). In this trimeric intermediate, PS-holoprotein likely adopts a conformation in which the hydrophobic domain that contains the site of PS-holoprotein endoproteolysis is membrane-embedded in a close proximity to TMD6 and TMD7, with the consequence of occluding the active site. The association of PEN-2 in the ER/cis-Golgi compartments to the trimeric and inactive iNCT/APH-1/PS-holoprotein subcomplex triggers a conformational change (in PS-holoprotein?) that promotes (1) the endoproteolytic cleavage of PS-holoprotein and subsequently (2) the activation and trafficking of γ-secretase, with (3) the conversion from an immature to a mature form of NCT (mNCT) with a different and more complex glycosylation pattern. The fully matured and active γ-secretase (mNCT/APH-1/PS-NTF/PS-CTF/PEN-2) is mainly localized in different cell compartments including the Golgi, the trans-Golgi network (TGN), the endosomes, the lysosomes and the plasma membrane.

**Fig. (4):**
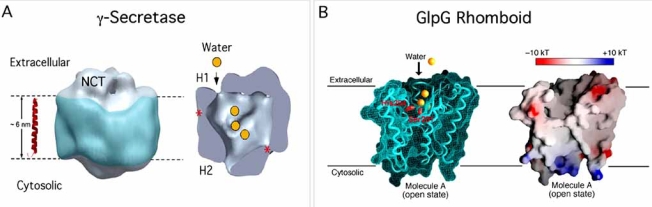
**Three-dimensional structure of γ-secretase and GlpG, an  *E.coli* intramembrane protease of the Rhomboid family. (A)** Electron microscopic three-dimensional representation of the γ-secretase complex at a resolution of 15 Å. Left: The potential transmembrane segment with the belt-like structure (blue) is outlined by two parallel dashed lines, 60 Å  apart, representing the membrane bilayer. The NCT ectodomain is located at the top density region as confirmed by lectin binding experiments. Right: A cut-open view of the complex from the side reveals a large central chamber of 20-40 Å in length, one opening (H1, 20 Å) facing the extracellular region and one opening facing the cytosolic region (H2, 20 Å). Two weak density regions are labeled with stars. Figure modified from [22] with permission (copyright National Academy of Sciences, USA). (**B**) Crystal structure of GlpG a bacterial rhomboid, in an open conformation and at a resolution of 2.6 Å. The putative catalytic-dyad Ser 201 and His 254 residues are shown in red. The water molecules (orange spheres) required to accomplish peptide bond hydrolysis may enter the membrane-embedded active sites by different routes from the outside (and/or inside?) aqueous solution, including the central chamber (GlpG) and the H1 and/or H2 pores (γ-secretase). Figure modified from [27] with permission.

**Fig. (5):**
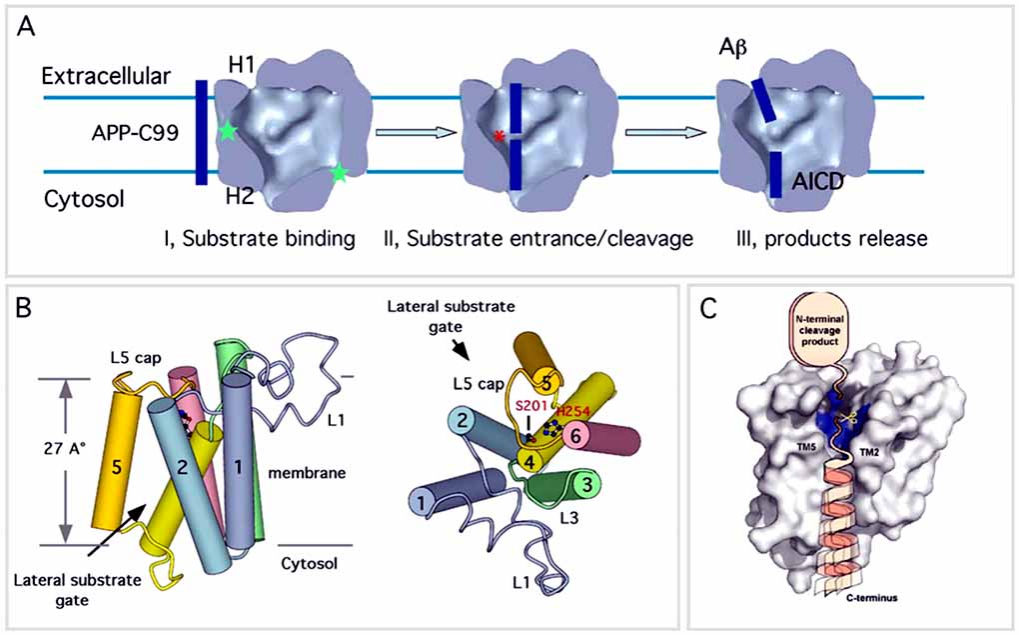
**Possible mechanisms of γ-secretase and GlpG rhomboid-catalyzed intramembrane proteolysis. (A)** Model for the γ-secretase dependent intramembrane processing of membrane proteins. In this model, the observed interior chamber and apical and basal pores (H1 and H2, respectively) would allow the entry of water molecules and their sequestration from the lipid surround by the proteinaceous microenvironment provided by the many transmembrane domains. The two thin-density regions in the transmembrane portion (labeled with green stars) may represent sites which could be opened up transiently (lateral gate-opening mechanism?) to enable entrance of the α -helically conformed substrates, i.e., the TMDs of various single transmembrane proteins (step I = substrate binding). After a substrate including APP-C-terminal fragments of APP (APP-C99 and APP-C83 with APP-C99 being only represented here) enters the water-filled central chamber, it undergoes hydrolysis by the two aspartate-containing catalytic site (red star) in PS (step II = substrate entrance/cleavage). The cleavage products that include the extracellular half of the substrate’s TMD (Aβ) could then be released through the H1 channel to the extracellular surface, whereas the product that includes the cytoplasmic half of the substrate’s TMD plus the C-terminal-intracellular domain (AICD for APP-IntraCellular Domain) could be released through the H2 channel to the cytoplasm (step III = products release). This possible mecha-nism further offers an explanation about how γ-secretase releases its two cleavage products into distinct subcellular compartments. (**B**) Side (left) and top (right) views of GlpG in an open cap conformation. The transmembrane helices are shown as cylinders labeled 1-6. A membrane-embedded loop structure (called L5 cap) is intrinsically flexible, consistent with the expectation that it can be displayed by the substrates, thus promoting the passage between TMD5 and TMD2 and active site access (proposed lateral substrate gate). The catalytic Ser 201 and His 254 residues are highlighted in red. Figure modified from [147] with permission (copyright National Academy of Sciences, USA). (**C**) A model for the interaction of substrates with GlpG (side view). TMD5 and TMD2 regions may represent sites which could be opened up transiently (lateral gate-opening mechanism) to enable entrance into the active site cavity of the α-helically conformed membrane substrate (straight or tilted) with the cleavage site being represented in a non-helical and accessible conformation that favors the proteolytic processing. Figure modified from [28] with permission (copyright National Academy of Sciences, USA).

## ABBREVIATIONS

Aβ: = Amyloid-β protein
AD: = Alzheimer’s disease
APP: = amyloid-β precursor protein
AICD: = APP intracellular domain
CHO: = Chinese hamster ovary
CTF: = C-terminal fragment
ER: = Endoplasmic reticulum
FAD: = Familial Alzheimer’s disease
HMW: = High molecular weight
IP: = Immunoprecipitation
kDa: = kiloDalton
NCT: = Nicastrin
NTF: = N-terminal fragment
PS: = Presenilin
TACE: = Tumor necrosis factor α (TNF-α)-converting enzyme
TGN: = Trans-Golgi network
TM: = Transmembrane
TMD: = Transmembrane domain
WT: = Wild-type
